# Light-based 40 Hz sensory therapy for brain disorders: physiological basis, therapeutic mechanisms, and future prospects

**DOI:** 10.3389/fmed.2026.1730333

**Published:** 2026-04-27

**Authors:** Chenyu Zhao, Xuran Peng, Zhi Cheng, Sanya Yue, Zikang Wang, Litian Ma, Jing Li, Mengjuan Shang

**Affiliations:** 1 Department of Radiation Protection Medicine, Faculty of Preventive Medicine, Fourth Military Medical University, Xi’an, China; 2 Ministry of Education Key Lab of Hazard Assessment and Control in Special Operational Environment, Fourth Military Medical University, Xi’an, China; 3 The Second Brigade of Student Affairs, Basic Medical College, Fourth Military Medical University, Xi’an, China; 4 The Fifth Brigade of Student Affairs, Basic Medical College, Fourth Military Medical University, Xi’an, China; 5 Department of Thoracic Surgery, Tangdu Hospital, Fourth Military Medical University, Xi’an, China; 6 Department of Traditional Chinese Medicine, Tangdu Hospital, Fourth Military Medical University, Xi’an, China; 7 Shaanxi Provincial Key Laboratory of Integrated Traditional Chinese and Western Medicine in Oncology Diagnosis and Treatment, Xi’an, China

**Keywords:** 40 Hz flickering light, 40 Hz flickering light and sound intervention, gamma oscillations, neuromodulation, neurological disorder, physiological mechanism, therapeutic mechanism

## Abstract

In recent years, 40 Hz flickering light and/or sound therapy has been confirmed to have certain therapeutic effects on Alzheimer’s disease (AD), although the underlying mechanisms remain unclear. This approach has been widely explored for the treatment of various neurological disorders, but its efficacy must be verified. The induction of gamma oscillations in the brain by 40 Hz flickering light and/or sound stimulation is likely a critical component underlying its therapeutic effects across brain diseases. Elucidating the physiological basis and mechanisms by which such stimuli induce gamma oscillations may reveal its mechanisms of action in treating diseases. Although 40 Hz flickering light and/or sound intervention offers certain advantages in improving neurological function, challenges related to technical optimization and clinical promotion must be addressed. Therefore, in this paper, the underlying mechanisms through which 40 Hz flickering light and/or sound intervention induces gamma oscillations, including both neuronal and non-neuronal mechanisms, are explained. The clinical therapeutic outcomes of 40 Hz flickering light and/or sound intervention for various neurodegenerative diseases are subsequently examined, and the mechanisms underlying are summarized. Furthermore, the limitations of this therapy and corresponding improvement measures are discussed, providing a theoretical reference for further refining this technology and expanding its clinical applications. Finally, future development directions are provided, with the aims of advancing related research and facilitating the application of this therapy in the treatment of brain diseases.

## 1 Introduction

Neural oscillations are rhythmic electrical activities generated by the synchronous activity of large populations of neurons in the central nervous system, arising from the properties of different cell types (including excitatory and inhibitory neurons) and their interactions (1–3). Among these, gamma oscillations are rhythmic electrical activities in the 30–90 Hz range produced by the synchronous firing of local neuronal populations. They are generated by the interaction between excitatory neurons and fast-spiking parvalbumin (PV) inhibitory interneurons and can be detected using techniques such as local field potential (LFP) and electroencephalography (EEG) (3). Gamma oscillations enable important excitatory signals within neural networks to escape inhibition and activate postsynaptic neurons within the temporal window of inhibition (4). The signal-to-noise ratio is enhanced by phase synchronization, during which the firing activities of different neuronal populations are aligned to the same rhythmic cycle (5, 6), thus promoting the pulsed transmission of stimuli (7), and generating preferential firing in response to preferred stimuli (8). This process, in turn, improves the coordination and precision of communication across multiple brain regions (4).

Under physiological conditions, endogenous gamma oscillations in the brain are closely associated with cognitive processes such as sensory information processing, memory encoding, and attention regulation (3, 4). Abnormalities in neural oscillations, particularly gamma oscillations, are observed in various neurological disorders. Among the different frequencies of gamma oscillations, 40 Hz has been the most extensively studied (9). Researchers such as Cardin and Sohal have proposed that this frequency matches the intrinsic oscillation frequency of PV inhibitory interneurons, enabling propagation through the resonant properties of cortical neural circuits and maintaining cross-regional coherence (10, 11). Numerous studies have confirmed that 40 Hz sensory stimulation can synchronize neural activity, restore gamma oscillations, and ameliorate pathologies and symptoms in various brain disorders. Owing to its many advantages, such as being safe, non-invasive, cost effective, and easy to implement, this approach has attracted considerable attention from the scientific community (12). Among these approaches, therapies based on flickering light, specifically, 40 Hz visual stimulation (VS) combined with 40 Hz audiovisual stimulation, have been the most widely investigated (13). Therefore, this review focuses on the role of 40 Hz flickering light and/or combined audiovisual stimulation in inducing gamma oscillations for the treatment of clinical disorders. The physiological basis of gamma oscillations induced by 40 Hz VS/VAS, the relationship between abnormal gamma oscillations and neurological diseases, and the current applications and limitations of 40 Hz VS/VAS in various brain disorders, with a focus on Alzheimer’s disease (AD), are summarized. The analysis highlights the advantages of this approach in improving neural function and the challenges remaining for its clinical translation. This review provides a theoretical foundation for the further development of this technology in the field of neuroscience, outlines future research directions, and aims to promote the optimization and clinical application of 40 Hz VS/VAS techniques.

## 2 Mechanisms of gamma neural oscillation induction by 40 Hz VS/VAS

### 2.1 Neuronal mechanisms

During gamma oscillations, rhythmic synaptic inhibition within local neural circuits regulates the firing activity of numerous neurons. With respect to the mechanisms underlying gamma oscillation generation, M.A. Whittington and colleagues proposed the pyramidal–interneuron network gamma (PING) model, which involves interactions between excitatory pyramidal neurons and inhibitory interneurons, and the interneuron network gamma (ING) model, which arises solely from interactions among inhibitory interneurons (14).

According to the PING model, the firing of excitatory neurons triggers the synchronous discharge of a large population of interconnected interneurons. This phenomenon leads to feedback inhibition that temporarily suppresses excitatory neurons, resulting in a brief resting period. As this inhibition subsides, the excitatory neurons fire again, initiating a new cycle of gamma oscillation. During the process of gamma oscillation induction by flickering light stimulation, when the retina receives rhythmic light stimuli, ganglion cells transmit visual signals via glutamatergic synapses to the lateral geniculate nucleus (LGN). Neurons in the LGN then relay excitatory signals via glutamatergic synapses to pyramidal neurons (PYR) in the primary visual cortex (V1). These PYR excite various interneurons, including PV, somatostatin (SST), and vasoactive intestinal peptide (VIP) interneurons, primarily through α-amino-3-hydroxy-5-methyl-4-isoxazolepropionic acid receptors. Upon activation, these interneurons, particularly PV+ cells, provide rapid feedback inhibition to pyramidal cells via γ-aminobutyric acid type A receptors, establishing regular gamma oscillations (15). Concurrently, PV, SST, and VIP inhibitory interneurons mutually inhibit each other, enabling flexible and dynamic regulation of real-time interactions within the local neural circuit (16).

In the ING model, interactions and synchronous firing among inhibitory interneurons induce synchronized inhibitory postsynaptic potentials in postsynaptic neurons, thereby generating gamma oscillations. Similar to the PING mechanism, when exogenous flickering light stimulation is applied, retinal ganglion cells transmit signals via glutamatergic synapses to the LGN. The LGN then directly transmits signals to inhibitory interneurons such as PV+ cells. A population of these interneurons begins to fire synchronously, generating synchronized inhibitory postsynaptic potentials in downstream neurons. The interactions within this network of inhibitory interneurons are sufficient to produce gamma oscillations (3, 15, 17).

Furthermore, on the basis of the influence of the membrane properties of inhibitory interneurons on oscillation frequency stability, Moca et al. proposed a novel resonant interneuron network gamma (RING) mechanism. The core of this mechanism lies in the membrane resonance properties (as opposed to the integration properties) of inhibitory interneurons. By reducing phase delays between interneurons and constraining the oscillation cycle duration, this resonance generates gamma oscillations with high frequency stability and power. The RING mechanism can also replicate the features of both PING and ING, thereby providing a significant theoretical advancement in the exploration of gamma oscillation mechanisms (18).

### 2.2 Non-neuronal mechanisms

Previous research on the mechanisms underlying gamma oscillation generation has focused primarily on neurons, with the role of glial cells often overlooked. However, direct evidence indicates that blocking vesicular release from astrocytes attenuates 30–50 Hz gamma oscillations in the mouse neocortex, confirming that astrocytes regulate fast gamma oscillations in neural circuits via vesicular release (e.g., of glutamate) (19). Additionally, activated microglia can increase the synchronous firing of cortical neurons in the gamma frequency band by migrating toward inhibitory synapses and removing them from cortical neurons (20). These findings suggest that non-neuronal cells also play a significant role in gamma oscillations. How, then, are glial cells activated by external frequency stimulation and subsequently participate in gamma oscillations? With respect to the activation mechanisms of non-neuronal cells in gamma oscillation generation, Adaikkan et al. proposed three hypotheses for glial cell activation. First, glial cells may directly participate in the generation of gamma rhythms by actively regulating ion flow between neurons through their ion buffering capacity. Second, glial cells might be passively involved in induced gamma oscillations, either through physical contact with neurons or by being passively activated via ionic fluctuations in the environment caused by neural oscillations. Third, glial cell functional states may be altered in response to modulation by neurotransmitters (21). Once activated, by what mechanisms do glial cells participate in gamma oscillations, and what specific roles do they play?

Astrocytes can modulate gamma oscillations through multiple mechanisms. The peripheral processes of astrocytes closely envelop glutamatergic synapses, acting as a physical barrier to restrict the diffusion of glutamate from the synaptic cleft. They also take up excess glutamate via glutamate transporters such as glutamate transporter 1 and glutamate-aspartate transporter, limiting glutamate spillover and reducing the activation of extrasynaptic N-methyl-D-aspartate receptors (22). Studies have shown that astrocytic calcium elevation, triggered by neuronal activity, can induce ATP release, which modulates the excitability and conduction velocity of myelinated axons and may influence the synchronization of gamma oscillations (23). Simultaneously, astrocytes can release the gliotransmitter S100B; its binding to the receptor for advanced glycation end products on neurons enhances kainate-induced gamma oscillations (24). Furthermore, astrocytes possess ion buffering capacity, which helps maintain the stability of the neuronal extracellular environment. They act as a chloride reservoir to regulate inhibitory networks by taking up chloride ions via the Na^+^-K^+^-2Cl^–^ cotransporter and releasing them through GABAA receptor activity (25). Additionally, through intracellular calcium transients, they actively modulate ion homeostasis by taking up extracellular potassium via Na^+^ and K^+^-ATPase, thereby influencing neural activity (26). Through the aforementioned mechanisms, astrocytes maintain the “excitation−inhibition balance” and the “homeostasis of the extracellular microenvironment” required for the generation of gamma oscillations, thereby directly or indirectly supporting the normal production, synchronization, and functional expression of gamma oscillations.

Microglia make direct contact with synapses (27). They can inhibit neuronal network hyperexcitability through the “ATP–ADP–adenosine” pathway and expand their surveillance territory via the “norepinephrine-β2 adrenergic receptor (β2R)” pathway, thereby helping to maintain the excitation–inhibition balance of neurons, which may play a significant role in gamma oscillations (28). In cerebral organoids, human induced pluripotent stem cell-derived microglia-like cells increase the power of gamma oscillations (30–100 Hz) through mechanisms such as the selective pruning of less active synapses, strengthening of functional synapses, regulation of ion channel expression, and coordination of the excitatory–inhibitory balance (29).

Myelination plasticity plays a significant role in neuronal oscillations (30). Oligodendrocytes participate in the formation of myelin and influence the generation of gamma oscillations. In studies on neuroligin-3-mutant mice, deficits in oligodendrocyte generation and myelination led to abnormal gamma oscillations (31). Similar conclusions have been reached in a cuprizone-treated mouse model of myelin injury, in which cuprizone toxicity induces oligodendrocyte loss, resulting in the demyelination of PV neurons, weakened PV inhibition, and subsequent hyperactivation of pyramidal cells, thereby disrupting neural synchronization (32). Furthermore, oligodendrocytes surrounding neurons that are consistently engaged in gamma rhythms likely respond to neuronal activity through “adaptive myelination,” thereby supporting the maintenance of gamma oscillations (33).

**FIGURE 1:**
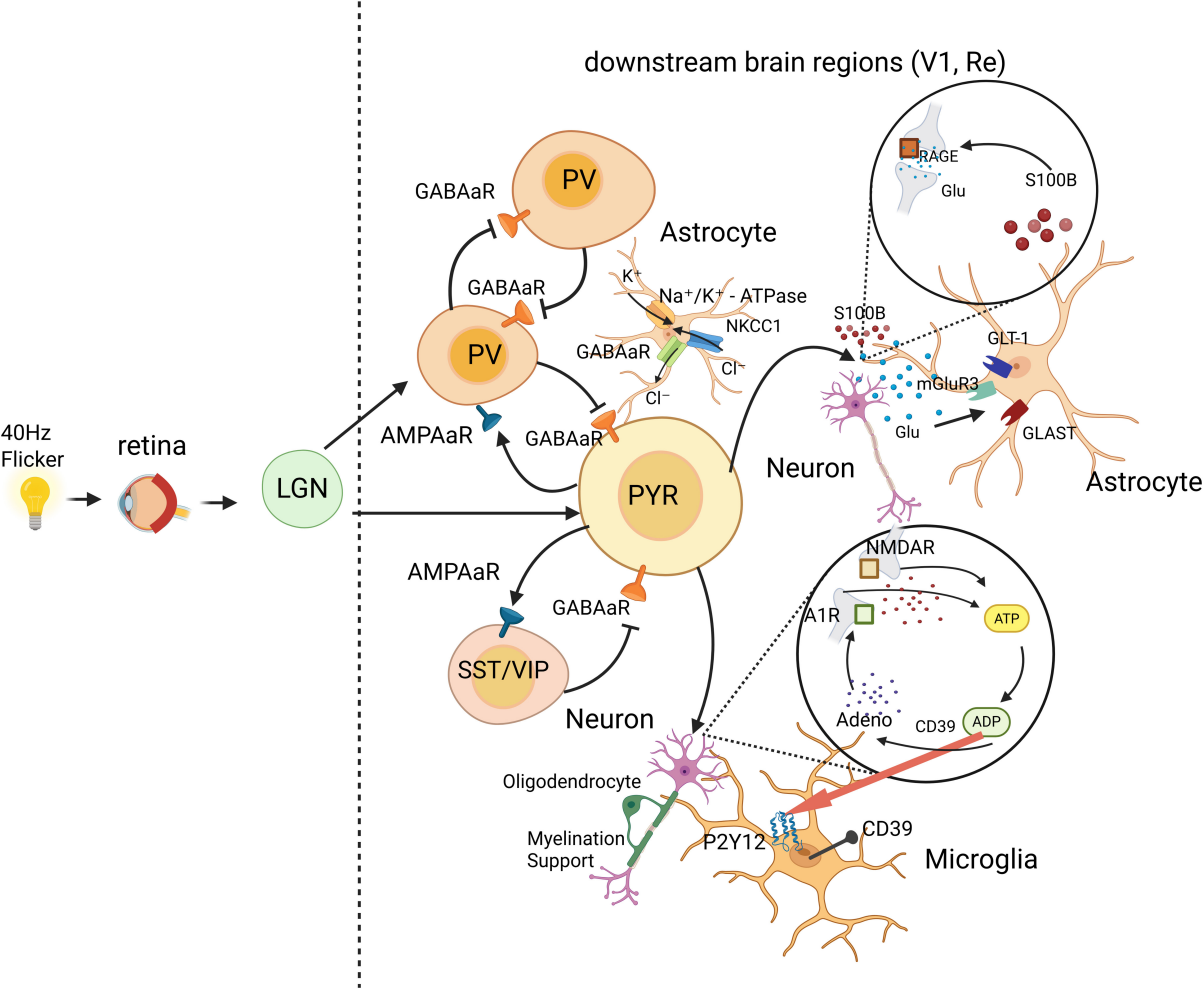
Neural mechanisms of 40 Hz-lnduced gamma oscillations in the brain. The information from 40 Hz flickering light stimulation is transmitted via the retinothalamic pathway to downstream brain regions, such as the visual cortex. This drives local PYRs and inhibitory interneurons to engage in the PING and ING interaction networks, thereby synchronizing neuronal activity in the downstream regions at the gamma rhythm (15). Astrocytes help maintain the “excitation-inhibition balance” and stabilize the extracellular microenvironment necessary for gamma oscillation generation by regulating neurotransmitter homeostasis, ion concentrations around synapses, and synaptic activity (22, 24, 25). Microglia inhibit neuronal hyperexcitability by acting on presynaptic N1 receptors through the “ATP-ADP-adenosine” pathway (28), whereas oligodendrocytes provide myelination support to sustain highly active neurons. The coordinated actions of these neural cells collectively contribute to the induction of gamma oscillations by 40 Hz flickering light stimulation (33). Created with BioRender.com.

In summary, the mechanism by which glial cells participate in gamma oscillations is likely activated after the induced synchronization of neuronal activity. Specifically, 40 Hz rhythmic sensory stimulation activates visual/auditory receptors, converting sensory information into electrical signals that are transmitted via neurons and synapses. This process synchronizes the electrical activity of neurons in various brain regions along sensory pathways, as well as in other regions connected by neural fibers. The activation of glial cells may thus be secondary to the rhythmic activity of neurons, representing an indirect regulatory effect of 40 Hz rhythmic sensory stimulation. Nevertheless, once activated, glial cells can participate in and modulate neuronal activity through multiple mechanisms (Figure 1). Therefore, glial cells may not merely be involved in secondary responses or be byproducts of neuronal activity and may instead play critical roles in such processes.

Notably, in human studies based on scalp electroencephalography (EEG), 40 Hz audiovisual stimulation can effectively induce the entrainment of gamma rhythms in the cerebral cortex, which is typically characterized by an increase in EEG oscillatory power. However, when interpreting these findings, it is imperative to consider the inherent inverse problem of EEG signal analysis (34, 35). This problem refers to the fact that the distribution of potentials recorded from the scalp cannot be uniquely inverted to precisely determine the location, orientation, and strength of the intracranial neural sources that generate them (35). This implies that gamma rhythms measured at the scalp level, which may appear similar, could originate from distinct neural generators within the brain, underpinned by completely different cellular mechanisms. Consequently, while the observed increase in 40 Hz oscillatory power based on scalp EEG confirms the overall modulatory effect of the stimulation on global brain activity, caution must be exercised when inferring its specific neural origins or a unified underlying mechanism of action. Furthermore, it is important to clarify that this “inverse problem” primarily constrains the interpretation of signals from non-invasive scalp EEG. In contrast, the animal intracranial electrode recording technique employed in animal studies, which directly localizes the recording site to a target brain region, allows for relatively clear identification of the neural sources of the measured gamma oscillations, thereby largely circumventing the ambiguity associated with signal source estimation. Future research should integrate high spatial resolution technologies such as functional magnetic resonance imaging (fMRI) or magnetoencephalography (MEG), or apply source localization algorithms [e.g., eLORETA (36)], to provide more direct anatomical evidence for the mechanisms of 40 Hz stimulation within specific brain networks.

## 3 Abnormal 40 Hz gamma oscillations and brain disorders

### 3.1 Neurodegenerative disorders

Neurodegenerative diseases are a class of disorders characterized by the progressive loss of neurons or myelin, accompanied by the pathological hallmark of abundant insoluble protein inclusions. Examples include the deposition of β-amyloid protein (Aβ) and tau proteins in AD and α-synuclein aggregates in Parkinson’s disease (PD). These protein aggregates exhibit neurotoxicity and may lead to neuronal death through mechanisms such as disruption of synaptic structures, induction of endoplasmic reticulum and mitochondrial dysfunction, and activation of inflammatory responses (37). The consequent disruption of neural network function due to neuronal death can further lead to abnormalities in neuronal activity, particularly gamma oscillations (38–40). PV and SST interneurons play critical roles in the generation of gamma oscillations. Optogenetic inhibition or activation of these interneurons has been shown to significantly reduce the power of cholinergically induced slow gamma oscillations. Therefore, any pathological factors that cause damage to these specific interneuron populations are likely to disrupt gamma oscillations (41). Furthermore, evidence suggests that a reduction in SST-positive/calretinin -positive interneurons in the lateral entorhinal cortex may contribute to abnormal gamma oscillations, indicating that the frequency and power of cortical gamma oscillations are likely coregulated by distinct neural circuits and different subpopulations of interneurons. Hence, any pathological factors capable of damaging these specific types of neurons are likely to disrupt gamma oscillations (42).

Abnormal gamma oscillations have been observed in various AD animal models and in AD patients. In animal studies, the phase locking value of high gamma rhythms and coherence with theta rhythms between the hippocampal perforant path and the dentate gyrus (DG) in 5xFAD mice were reduced, accompanied by decreased expression of GABA Rα1 (38). APP/PS1 mice exhibited increased power of low gamma oscillations (40–70 Hz) in the olfactory bulb (OB), which coincided with the onset of olfactory deficits (43). In 3xTg AD mice, a reduction in slow gamma wave power occurred even in the prepathological stage (prior to Aβ deposition) (44), suggesting that abnormal gamma oscillations may serve as early markers of AD (45). Evidence from human studies mirrors observations in AD mouse models, gamma oscillation abnormalities are present in the early stages of AD (46) and may even precede the excessive accumulation of Aβ (39). Gaubert et al. conducted electroencephalogram (EEG) and positron emission tomography (PET) scans on 314 AD patients and reported an inverted U-shaped curve relationship between gamma oscillation power and amyloid deposition: the power increased with low to moderate amyloid levels but decreased after the critical threshold was exceeded. This phenomenon suggests that low to moderate amyloid levels may trigger “compensatory activation” in the brain, which fails under high amyloid burden, leading to a shift toward pathological brain electrical activity (47).

Patients with Parkinson’s disease present clinical manifestations, including motor dysfunctions such as resting tremors and bradykinesia, in addition to non-motor symptoms such as cognitive impairment and depression (48). In the brains of PD patients, abnormal intracellular aggregation of α-synuclein results in the formation of Lewy bodies, which can interfere with neuronal physiological functions, leading to cellular damage and death. Its core pathogenesis involves the progressive loss of dopaminergic neurons in the substantia nigra pars compacta, resulting in dysfunctional basal ganglia circuitry. This dysfunction is characterized by hyperactivity of the indirect pathway (which suppresses movement) and hypoactivity of the direct pathway (which promotes movement), disrupting the balance between movement-suppressing beta-band activity and movement-promoting gamma-band activity. This imbalance is accompanied by the emergence of abnormal transient high-frequency gamma oscillations (49). Motor dysfunction in PD patients is closely associated with this imbalance in neural oscillations. With respect to animal studies, Zemel et al. reported that in mice, dopamine deficiency led to abnormally enhanced synchrony within networks of medium spiny neurons in the striatum, coupled with reduced motor encoding capability of PV interneurons and the appearance of transient pathological LFP high-gamma oscillations. This abnormal state of the striatal neural network contributes to motor dysfunction (49).

### 3.2 Psychiatric and neurological disorders

Psychiatric and neurological disorders include conditions such as depression, bipolar disorder (BD), schizophrenia, and autism. Gamma oscillations are associated with abnormalities in multiple psychiatric and neurological diseases and are therefore regarded as potential clinical biomarkers (9). Clinical trials in humans have shown that major depressive disorder (MDD) is typically characterized by persistent low mood or dysthymia. Its pathological mechanisms are complex and involve various hypotheses, including the monoamine neurotransmitter hypothesis and the inflammation hypothesis (50). Clinical studies have revealed abnormalities in the frequency spectrum of EEG signals in MDD patients, indicating impaired neuronal network function (51). Among these findings, gamma power within the limbic system and its long-range desynchronization serve as potential biomarkers for MDD patients (52). Deep brain stimulation applied to the ventral striatum/ventral septal nucleus in MDD patients has been shown to reduce gamma power in the amygdala and alleviate depressive symptoms (53). Experimental studies on rodents have revealed the OB as a key pacemaker driving gamma oscillations in the limbic system (54, 55). The inhibition of OB neuronal activity significantly reduces gamma oscillation power both locally and in downstream limbic regions such as the piriform cortex, subsequently inducing depression-like behaviors, including anxiety and anhedonia. The severity of these behavioral abnormalities is positively correlated with the extent of the reduction in gamma oscillation power in the PirC. These findings suggest that identifying the source brain regions responsible for abnormal gamma oscillations in MDD and developing targeted modulation strategies may hold significant therapeutic potential (56).

Bipolar disorder is a highly heritable psychiatric illness. Although genetic factors play a central role, other mechanisms, including neurotransmitter imbalances and neuroinflammation, are likely involved; however, its precise pathogenesis remains incompletely understood (57, 58). BD is characterized by the concurrent or alternating occurrence of manic and depressive episodes, often accompanied by sleep disturbances and abnormal neural oscillations (59). Clinical studies have shown that in patients with BD, abnormalities in the generation and maintenance of intrinsic gamma oscillations (30–90 Hz) occur within the reticular activating system, particularly those mediated by voltage-dependent calcium channels in the pedunculopontine tegmental nucleus (PPN) (60–62). The overexpression of neuronal calcium sensor-1 severely impairs the ability of PPN neurons to generate and maintain gamma oscillations during wakefulness by inhibiting the function of P/Q-type calcium channels. This impairment directly contributes to symptoms in patients with BD, such as cognitive deficits, difficulties with perceptual binding, and disruptions in the stream of consciousness, which occur despite a state of hyperarousal (59, 63).

Autism spectrum disorder (ASD), schizophrenia, and other psychiatric disorders share a common pathological mechanism: abnormalities in the function and morphology of inhibitory interneurons expressing PV, particularly the chandelier cell subtype (64, 65). PV-positive neurons are central to cortical inhibitory microcircuits and form the cellular basis for driving and maintaining cortical gamma oscillations (30–100 Hz) (11, 66). These neurons exhibit high metabolic rates, rendering them vulnerable to damage from factors such as oxidative stress, which can lead to apoptosis and dysfunction (67, 68). This process results in disrupted GABAergic signaling, including reduced expression of glutamate decarboxylase, key enzyme for presynaptic GABA synthesis (69, 70), and alterations in the subunit composition (e.g., α_2_) of postsynaptic GABAA receptors (71, 72), ultimately disrupting the cortical excitation/inhibition balance and causing abnormalities in gamma oscillations. Clinical studies have revealed that patients with schizophrenia exhibit abnormalities in both spontaneous and evoked gamma oscillations, and these aberrant gamma oscillations are associated with clinical symptoms (73).

### 3.3 Sleep disorders

The core pathological mechanism of sleep disorders stems from an imbalance in the cellular pathways regulating wakefulness and sleep within the PPN. The PPN regulates wakefulness and rapid eye movement (REM) sleep through two distinct intracellular pathways: the “wakefulness” pathway, which is governed by calcium/calmodulin-stimulated protein kinase II (CaMKII) modulation of P/Q-type calcium channels, drives gamma oscillations during wakefulness. In contrast, the “REM sleep” pathway, which is regulated by protein kinase A (PKA) system modulation of N-type voltage-dependent calcium channels, drives gamma oscillations during REM sleep (59, 61, 62). Hyperactivity of the CaMKII-P/Q pathway causes the neuronal population containing P/Q-type channels within the PPN to remain dominant during sleep periods, thereby inhibiting the initiation and maintenance of sleep (59, 74). This pathway imbalance directly manifests as the persistent generation of abnormal gamma oscillations by PPN at night, which maintains the cerebral cortex in a hyperaroused state, disrupting the normal sleep–wake cycle, and clinically presents as difficulty falling asleep, sleep maintenance problems, and a reduced total sleep time in patients with insomnia (75).

Sleep disorders are not only a distinct category of illness but are also prevalent in various other neurological diseases and may even drive their pathological processes in the early stages (76). For example, sleep disturbances in patients with AD can manifest in several ways. Patients with AD exhibit a loss of neurons in the suprachiasmatic nucleus (SCN), abnormal expression of melatonin receptors, and loss of retinal ganglion cells, which can lead to dysregulation of the circadian rhythm. Moreover, disruption of circadian rhythms can inhibit the glymphatic system, reduce Aβ clearance, and exacerbate neuronal damage, and thus accelerating the progression of AD (77). Such secondary sleep disorders also involve abnormal neural oscillations. Moguilner et al. reported that patients with AD without comorbid epilepsy show abnormally enhanced functional connectivity in the gamma frequency band within the frontotemporal regions during non-rapid eye movement (NREM) stage 2 sleep. This abnormality is closely associated with longitudinal cognitive decline (78).

### 3.4 Brain injury disease

Stroke, also known as cerebral apoplexy, is an acute cerebrovascular disease. Its fundamental pathological changes involve cerebrovascular lesions, hemodynamic abnormalities, and abnormalities in blood components, which collectively lead to neuronal necrosis and damage to brain tissue (79). Multiple studies on rodents have demonstrated the presence of abnormal gamma oscillations in the brains of mouse models of stroke. In a mouse model of unilateral hippocampal ischemia, gamma oscillations in the ipsilateral hippocampus were persistently reduced following high-frequency discharges (80). In the Tamura model (a mouse model of permanent cortical ischemia), the power of low gamma oscillations (30–50 Hz) in the peri-infarct cortex continuously decreased, with the extent of the decrease showing a strict gradient relationship to the distance from the ischemic core (81). In a cerebral ischemia model induced by bilateral common carotid artery occlusion (two-vessel occlusion, 2VO), reduced power of slow gamma oscillations in the CA1 region and disrupted theta–low gamma phase–amplitude coupling were detected (82). In human studies, the abnormal spatial distribution of cortical gamma synchrony may be related to anatomical structural damage in stroke patients. Furthermore, auditory-evoked gamma synchrony can serve as a reliable biomarker for assessing the clinical status of stroke patients and effectively predict their functional outcomes (83). Lu et al. analyzed EEG data from 559 patients with poststroke aphasia across 31 studies and reported increased low-frequency EEG activity and decreased high-frequency EEG activity. Specifically, reduced gamma oscillation activity in the left hemisphere during delayed reading tasks was negatively correlated with aphasia severity. These abnormalities in neural oscillations disrupt functional connectivity within brain networks, thereby impeding the coordination and cross-regional integration of language information (84).

Furthermore, abnormalities in gamma oscillations have been observed in other types of brain injury and are closely associated with cognitive decline resulting from such damage. For example, traumatic brain injury leads to abnormalities in the excitability of hippocampal pyramidal neurons and in synaptic transmission (85), whereas chemotherapy-induced brain injury involves persistent dysregulation of microglia, astrocytes, and oligodendrocytes, as well as defects in myelination (86). Such impairments may further lead to gamma oscillation abnormalities and cognitive dysfunction.

### 3.5 Epilepsy

Epilepsy is characterized by unexpected and spontaneous recurrent seizures, with its core pathological mechanism being an “excitation–inhibition imbalance” within neuronal networks, leading to excessive synchronous discharges. In adult patients with epilepsy, a hyperexcitable inhibitory neural network can be triggered by minor physiological fluctuations, resulting in an abrupt transition from chaotic to synchronized firing and subsequently leading to enhanced activity (87). This excitation–inhibition imbalance is likely the intrinsic mechanism underlying abnormal gamma oscillations. Patients with epilepsy exhibit abnormal neural synchronization (88). During generalized seizures, the power of gamma oscillations and the coherence of neuronal activity within and between hemispheres are significantly greater than they are during interictal periods, which may be related to pathological network activity in idiopathic generalized epilepsy (89). Differences also exist between physiological and pathological high-frequency oscillations (HFOs) in the brains of patients with focal epilepsy; HFOs originating from the epileptogenic zone have higher amplitude, lower frequency, and longer duration (90). In studies on photosensitive epilepsy, gamma oscillations evoked by specific VS are significantly stronger in patients than in healthy controls, suggesting that the “neural circuits generating narrowband gamma oscillations” may have inherent stability deficits in these individuals (91).

## 4 Therapeutic efficacy and application potential of 40 Hz stimulation for various brain disorders

The capacity of 40 Hz sensory stimulation to modulate neural synchrony and restore gamma oscillations provides a compelling rationale for its therapeutic application. Research indicates that leveraging this approach can mitigate pathological hallmarks and alleviate clinical symptoms in diverse brain disorders, as detailed in the following subsections (Table 1).

**TABLE 1:** Therapeutic efficacy and application potential of 40 Hz stimulation for various brain disorders.

Disease type	Subject	Method	Therapeutic mechanism	Therapeutic efficacy	References
AD	Patients with MCI due to AD	40 Hz VAS,1 h/day, 4/8 weeks	Functional connectivity between PCC and PCu↑ Immune factors and cytokines↓	Engagement of 40 Hz neural activity No significant changes in CSF Aβ and tau levels Safe, tolerable, and feasible	(101)
	Patients with mild AD	Single session of 1 h 40 Hz VAS	Induce 40 Hz gamma entrainment acrosscortical sensory regions (frontal lobe, occipital lobe) subcortical regions (gyrus rectus, amygdala, hippocampus, insula).	Safe, tolerable, and feasible	(12)
	Patients with mild AD	40 Hz VAS 1 h/day 3 months	PCC and PFC, angular gyrus connection↑ Hippocampus and VC connection↑	Safe, tolerable, and feasible Ventricular enlargement↓ Hippocampal atrophy↓ Performance on an associative memory task↑ Markers of sleep↑	
	Patients with mild to moderate AD	40 Hz VAS 1 h/day, 6 months		Safe, tolerable, and feasible Sleep quality↑ Activities of daily living ability↑	(107)
	Patients with AD	40 Hz low-perceptible flicker, 5 h/day, 12 weeks	–	Safe, tolerable, and feasible No significant change in cognitive function Ability of daily living↑ Depression↓ Euphoria↑ Caregiver burden↓	(127)
	5XFAD mice	40Hz VAS 1 h/day, 8 days	CA3–CA1 slow gamma-band functional coupling↑ CA3–CA1 circuit signal transmission efficiency↑	Spatial memory encoding↑ Correlation among speed, task, expected behavior, and prospective coding↑	(102)
	Tau P301S mice CK-p25 mice	40 Hz VS, 1 h/day, 22 days/6 weeks	Microglial morphological transformation cytoprotective proteins ↑ Neuronal DNA damage↓, Synaptic function↑ Neuroinflammation↓ Functional coupling of V1–CA1 and V1–PFC↑	Number of neurons↑ Spatial memory↑ Anxiety↓γ oscillations in V1, CA1, PFC, and SS1↑	(103)
	5XFAD mice APP/PS1 mice Tau P301S mice	40 Hz VS, 1 h/days, 1–7 days	Microglial transformation into a phagocytic state APP processing↓ Neuroinflammation↓	Aβ↓,Phosphorylated tau↓γ synchronization among CA1, VC, and PFC↑	(111)
	APP/PS1 mice	40 Hz VS, 1 h/day, 30 days	Rhythmic firing in SCN neurons↑ Inhibitory synaptic transmission↑ Excitatory synaptic transmission↑ APP,β-CTF↓	Circadian rhythm↑ Aβ↓ Phosphorylated tau↓γ oscillations in VC↑	(148)
	5XFAD mice	40 Hz VAS, 1 h/day, 10 days	Astrocytic AQP4 polarization↑ Glymphatic function↑ VIP↑, arterial pulsatility↑	Spatial memory and cognitivediscrimination ability↑, Aβ↓γ synchronization among VC, Posterior frontal lobe↑	(122)
	5XFAD mice APP/PS1 mice Tau P301S mice	40 Hz VS, 1 h/day, 7 days	Transition of microglia to phagocytic state Astrocyte activation↑ Colocalization of LRP1 and Aβ↑	Spatial recognition memory ↑, Aβ↓γ synchronization among AC,VC,CA1,mPFC↑	(112)
	5XFAD mice	40 Hz VS, 1 h /day, 14 days	Activation of the vLGN/IGL-re visual circuit↑ AQP4 in astrocytes ↑ Lymphatic-like function ↑ Hippocampal lipid metabolism↓	Spatial working memory ↑ Long-term recognition memory ↑ Motivational behavior ↑ Aβ↓	(104)
	STZ-induced AD rats	40 Hz VS, 15 min/day, 7 days	Mitochondrial mitoBKCa channel function ↑ Mitochondrial respiratory chain function↑ Synaptic transmission and plasticity ↑ Restoration of metabolite balance Immune response ↑	Long-term recognition memory ↑ Spatial memory ↑ Cell apoptosis ↓ Aβ↓	(160)
	Aβ oligomer-induced AD mice	40 Hz VAS, 1 h/day, 4 weeks + involuntary or voluntary exercises	Activation of BDNF/TrkB/Akt pathway Promotion of proliferation and differentiation of aNSCs Improvement in glutamate metabolism, glucose metabolism, and TCA cycle Synaptic plasticity ↑ Astrocyte-clustering responses↑	Cognitive function ↑ Anxiety ↓ Depression ↓ Mature neurons in DG ↑γ oscillations in visual and auditory cortex ↑	(114)
	P301S mice	40 Hz VS, 1 h/day, 21 days	Activation of olfactory-related brain region neurons Inflammatory response ↓	Olfactory function ↑ No significant change in P-tau RNA	(106)
PD	PD mice	40 Hz VAS, 2 h/day, 1/6 months	Activation of sensory and motor cortex neurons	Motor and cognitive function ↑ Depression ↓α-Syn↓γ oscillations ↑	(123)
Depression	CORT mice CRS mice	40 Hz VS, 2 h/day, 21 days	Transition of microglia to anti-inflammatory state IL-12↓, Neuroinflammation ↓ Restoration of synaptic structure and function	Depression ↓, anxiety ↓ Spatial working memory ↑ Recognition memory ↑γ oscillations in V1, CA1, and PFC ↑	(126)
	Patients with mild MMD	40 Hz Violet flicker, 3 h /day, 4 weeks	–	Depression ↓ Safe No significant change in somatic Symptoms of MMD	(128)
	Patients with MMD	40 Hz non-flickering, light, 1 h /day, 6 weeks	This is a clinical experimental design with no research results yet		(129)
ASD	116p11.2 deletion mice	40 Hz VS 1 h /day, 14 days	Activation of adenosine - A1R pathway Excitatory neurotransmission ↓ Inhibitory neurotransmission ↑	Social novelty deficit ↓ Multiband γ power in PFC ↓ Safe	(140)
Sleep disorders	Transgenic mice Children with insomnia	40 Hz VS single session 30 min	Adenosine ↑ V1 neuronal activity ↓ Phosphorylation level of AMPK ↑	Sleep duration ↑ Normalization of sleep structure Acceleration of sleep initiation Postsleep awakening time ↓	(144)
	Patients with insomnia	40 Hz VS, 1 h /day, 8 weeks	–	Sleep onset latency ↓ Sleep time ↑ Sleep continuity ↑ Safe, tolerable, and feasible	(161)
	RD mice	Single session of 1 h 40 Hz VAS	–	Sleep-wake rhythm phase shift ↓ Deficits in fear conditioning memory↑	(149)
	72-h sleep-deprived rats	Single session of 1 h 40 Hz VAS	5-HT↑ Melatonin ↑ Cortisol ↓ Activation of Brain Regions Such as hippocampus, SCN	Spontaneous locomotor activity ↑ Spatial exploration and vigilance ↑ Thalamic γ oscillations↑ and θ oscillations↓	(150)
Stroke	2VO-induced global cerebral ischemia mice	40 Hz VS, 2 h/day, 14 days	CA3-CA1 excitatory synaptic transmission ↑ CA1 neuronal damage ↓	CA1 γ oscillations↑θ-Low γ coupling ↑ Locomotion, spatial learning and memory ↑	(82)
	RUCCAO-induced CCH mice	40 Hz VS, 1 h /day, 30 days	Neuroinflammation ↓ Cognition-related genes ↑ Neuronal Protection ↑	Short-term memory ↑ Spatial working memory ↑ Number of hippocampal neurons ↑ Anxiety ↑	(162)
	PSA mice	40 Hz VS, 2 h/day, 21 days	HDAC3/COX-1/EP2 pathway ↓ PGE2↓ Neuroinflammation ↓ Synaptic function ↑	Depression ↓ Anxiety ↓	(125)
	Acute anterior circulation ischemic stroke patients	40 Hz VS, 25 min/day, 14 days	This is a clinical experimental design with no research results yet		(154)
TBI	TBI mice	40 Hz VS, 2 h/day, 25 days	Postsynaptic excitability ↓ Neuronal damage ↓ PSD95 ↓ CA3-CA1 LTP↑	Spatial, working, and passive avoidance memory ↑γ oscillations ↑θ- low γ coupling ↑	(155)
Chmobrain	Chemobrain mice	40 Hz VAS, 1 h/day, 21 days	Chemotherapy resistance genes ↑ Apoptosis genes ↓ Myelin sheath thickness and number ↑	Anxiety ↓ memory ↑ Executive function ↑ Mature oligodendrocytes ↑ Microglia and astrocytes ↓γ oscillations ↑	(158)
Epilepsy	Amygdala kindling in 5xFAD/WT mice	40 Hz VAS, 1 h/day, 5 weeks	Neuroinflammation ↓ Astrocyte and microglial activation	Seizure severity ↓ Seizure susceptibility ↓ Aβ↓γ oscillations ↑	(152)
	Treatment-resistant epilepsy patients	5.5/40/80 Hz VAS/VS/AS, short time	Neuronal synchronous activity ↑	IEDs↓γ oscillations ↑ Safe, tolerable, and feasible	(153)
Demyelinati-ng disorder	Demyelinated mice	40 Hz VAS, 1 h/day, 3 weeks	Neuroinflammation↓ Ferroptosis inhibitors↑ Oligodendrocyte function↑ Suppression of microglia and hyperactivation of astrocytes	Restoration of myelin structure Myelinated axons↑γ oscillations↑	(159)

### 4.1 Efficacy of 40 Hz VS/VAS in neurodegenerative diseases

#### 4.1.1 Efficacy of 40 Hz VS/VAS in AD

Alzheimer’s disease is characterized by progressive cognitive decline, accompanied by emotional and behavioral abnormalities as well as psychiatric symptoms, which severely impair patients’ ability to perform activities of daily living. Current management of AD relies primarily on pharmacological interventions, such as cholinesterase inhibitors (e.g., donepezil, galantamine, and rivastigmine) and NMDA receptor antagonists (e.g., memantine). However, these treatments can alleviate only cognitive symptoms or slow disease progression; they cannot halt or reverse underlying pathological processes such as the formation of Aβ plaques and tau neurofibrillary tangles (92, 93). Furthermore, the blood–brain barrier significantly restricts the delivery of therapeutic agents to the central nervous system (94). Most interventions are initiated only after symptoms appear, at which time extensive neuronal damage has already occurred, and the optimal therapeutic window is missing (95). There is an urgent need to develop more effective novel treatments that target the fundamental pathological changes of AD. In recent years, 40 Hz visual/auditory stimulation (VS/VAS) has been demonstrated to promote the clearance of pathological proteins such as Aβ and improve functional connectivity in the brains of patients with AD. It has shown preliminary efficacy in mitigating cognitive decline and other accompanying symptoms, highlighting the promising application prospects of 40 Hz VS/VAS in treating neurodegenerative diseases (12, 96).

Brain connectivity serves as an indicator of synchronization between brain regions or the coordinated activity of different brain areas. Existing evidence from studies on human indicates that abnormal functional connectivity occurs in the brains of patients with AD and involves multiple regions, with the posterior cingulate cortex (PCC) serving as a seed area and the hippocampus as a core component (97). Mild cognitive impairment (MCI) related to AD manifests as widespread atrophy in core regions of the default mode network (DMN) and a significant weakening of internal functional connectivity, with the degree of abnormality correlated with disease progression (98, 99). Human studies have shown that 40 Hz VS/VAS can restore abnormal functional connectivity in the brains of AD patients, and similar effects have been observed in healthy individuals (12, 96, 100). He et al. administered 40 Hz audiovisual stimulation to 10 patients with MCI due to AD for 1 h daily over 8 weeks. Post-treatment, the strength of functional connectivity between the PCC and the praecuneus (PCu) significantly increased (101). Long-term combined 40 Hz audiovisual stimulation over 3 months enhanced functional connectivity within the DMN and the medial visual network (MVN) in patients with mild AD dementia (12). In another study, after treatment with combined 40 Hz audiovisual stimulation, patients with AD showed significantly enhanced functional connectivity between the PCC and key nodes of the DMN (12), further demonstrating that 40 Hz VS can increase neural synchrony within the DMN centered on the PCC. Notably, impairment of the DMN is a recognized pathological feature of AD (98). In healthy individuals, acute 40 Hz VS enhances functional connectivity between the dorsolateral prefrontal cortex (PFC) and the visual cortex (96), as well as between the hippocampus and the superior parietal lobe (100). From the perspective of brain topology, 40 Hz VS can result in the construction of an optimal functional network characterized by the “shortest global path + maximum local clustering,” enabling highly efficient neural information transfer (96). Researchers have extensively investigated the effects of 40 Hz sensory stimulation on functional connectivity in the brains of AD model mice. By recording the electrical activity of hippocampal neurons during spatial navigation tasks in AD mice, they reported that functional connectivity between the CA1 and CA3 regions within the hippocampus was increased. This specific neural activity is crucial for memory function (102). Further mechanistic studies have suggested that the enhancement of connectivity between different brain regions by 40 Hz visual stimulation may be achieved by synchronizing gamma oscillations across multiple regions, enhancing synaptic plasticity, and stabilizing synaptic connections (103).

Numerous studies have demonstrated that 40 Hz sensory stimulation can improve cognitive function and daily living ability in both AD animal models and AD patients. In animal studies, Wu et al. reported that after treatment with 40 Hz blue light, 4-month-old 5XFAD mice showed significant improvements in spatial working memory and long-term recognition memory. Moreover, 14-month-old 5XFAD mice, representing middle- to late-stage AD, exhibited reduced exploratory behavior, which was significantly ameliorated by blue light treatment. These findings suggest that 40 Hz VS applied during the early and mid-to-late stages of AD can improve cognitive function and motivational behavior, respectively (104). Olfactory dysfunction is a hallmark symptom that occurs in the early stages of AD and is important for understanding its pathophysiology and early diagnosis (105). A total of 40 Hz flickering light has been shown to improve olfactory function in AD model mice by modulating immune responses (106). In terms of human clinical research, Chan et al. administered combined 40 Hz audiovisual stimulation to patients with mild AD for 1 h daily for 3 months. Using the free and cued selective reminder test, they reported significant improvement in episodic memory in the treatment group, along with significantly higher scores related to the stability of daily activity rhythms (12). In another 6-month trial, the flickering light treatment group had significantly increased scores on the Alzheimer’s Disease Cooperative Study - Activities of Daily Living Scale, indicating improved daily living abilities (107). On the basis of a comprehensive analysis of 974 patients across 16 studies, Octary et al. concluded that multisensory stimulation (including combined audiovisual stimulation) can improve cognitive function and significantly reduce agitation, apathy, and depressive symptoms in patients with AD (108). Interestingly, the effects of 40 Hz VS on gamma oscillations and cognitive performance are sex dependent, with greater improvement in cognitive ability observed in males than in females (109).

Thus, 40 Hz stimulation demonstrates unique therapeutic efficacy for AD. However, through what mechanisms does it exert these therapeutic effects? A series of animal studies have been conducted to elucidate the underlying protective mechanisms. AD is characterized by two primary pathological hallmarks, amyloid plaques (Aβ deposition) and neurofibrillary tangles (tau protein deposition), which lead to secondary pathological changes, progressive neurodegeneration, and neuronal death (110). Research has shown that 40 Hz VAS can ameliorate these fundamental pathologies in AD. In various mouse models (including 5XFAD, APP/PS1, and WT mice), 40 Hz flickering light or combined audiovisual stimulation has been shown to reduce Aβ production and promote its clearance across multiple brain regions, decrease the levels of both soluble and insoluble Aβ, and reduce the number and size of amyloid plaques (111, 112). Martorell et al. administered synchronized 40 Hz audiovisual stimulation to P301S mice for 1 h daily for 7 days, which resulted in reduced phosphorylation levels of tau protein in these mice (112). Mechanistically, 40 Hz sensory stimulation can modulate the cleavage process of the amyloid precursor protein (APP), thereby reducing Aβ generation (111). In addition, such stimulation increases the number of microglia and promotes an activated phenotype, enhancing the ability to phagocytose Aβ. Moreover, this stimulation increases the number of reactive astrocytes and decreases blood vessel diameter, facilitating the clearance of Aβ across the vasculature (112). Furthermore, 40 Hz stimulation increases the polarization of the AQP4 water channel in astrocytes, promotes the dilation of meningeal lymphatic vessels, and activates VIP neurons to increase VIP secretion. This VIP secretion regulates arterial pulsations, providing the driving force for cerebrospinal and interstitial fluid flow. Through these combined actions, it promotes the clearance of metabolic waste and Aβ from the brain via the glymphatic system. Some studies also suggest that the adenosine-A2A receptor signaling pathway mediates the enhancement of glymphatic flow by 40 Hz VS, with increased astrocytic aquaporin 4 (AQP4) polarization and enhanced vasomotion being key components in this process (113). Concurrently, Wu et al. revealed the potential neural circuit basis underlying 40 Hz modulation of brain function. They reported that low-intensity 40 Hz blue light, by activating the vLGN/IGL-Re visual circuit, restores the perivascular polarization of astrocytic AQP4. This phenomenon enhances the cerebrospinal fluid (CSF) influx and interstitial fluid outflow functions of the glymphatic system, thereby promoting the clearance of soluble Aβ and lipids (104). Previous studies have indicated that 40 Hz flickering light therapy can protect mitochondrial function and improve synaptic plasticity by repairing the respiratory chain, modulating mitochondrial large conductance calcium-activated potassium channels (mitoBKCa), and restoring the metabolite balance. These mechanisms collectively alleviate pathological Aβ deposition in streptozotocin-induced AD models (104).

Furthermore, animal studies have shown that when combined with exercise therapy, 40 Hz VS has additional regulatory effects on neurogenesis. Li et al. reported that a multifactorial intervention combining 40 Hz audiovisual stimulation with both voluntary and involuntary exercise activated the brain-derived neurotrophic factor (BDNF)/tropomyosin receptor kinase B (TrkB)/protein kinase B (Akt) pathway. This intervention improves energy metabolism and neurotransmitter metabolism, repairs Aβ-induced adult neurogenesis deficits, and subsequently reverses cognitive impairment and anxiety/depression-like behaviors, with effects superior to those of any single intervention alone (114). Similarly, combining audiovisual stimulation with aerobic exercise and olfactory stimuli promotes adult neurogenesis through the BDNF pathway while suppressing oxidative damage via the cytoglobin pathway (115). Yan et al. identified a comparable mechanism in normal mice, whereby 40 Hz VS activates PV neurons to increase GABA release, thereby promoting neurogenesis (116).

#### 4.1.2 Therapeutic potential of 40 Hz VS/VAS for PD

The primary pathological features of PD include excessive deposition of neurotoxic α-synuclein and progressive degeneration of dopaminergic neurons in the substantia nigra pars compacta (116–118). Dopamine deficiency can lead to reduced power and frequency of gamma oscillations during movement in patients with PD, which may represent an underlying pathological mechanism for bradykinesia (119). Research has shown that a combined protocol of transcranial magnetic stimulation and transcranial alternating current stimulation (tACS) can enhance cortical gamma oscillations and restore impaired cortical LTP-like synaptic plasticity in patients with PD (120). In these patients, sleep disorders and impairment of AQP4 channels lead to decreased glymphatic system efficiency, hindering the clearance of waste products such as α-synuclein. The accumulation of α-synuclein, in turn, further disrupts AQP4 function, creating a vicious cycle (121). Concurrently, mitochondrial dysfunction contributes to reduced CSF production, exacerbating the weakening of glymphatic flow. These factors collectively promote the deposition of neurotoxic α-synuclein (121). Patients with PD have pathological features similar to those of patients with AD (110); moreover, the efficacy of 40 Hz audiovisual combined stimulation has been confirmed in AD (111). In addition, 40 Hz VS can activate the glymphatic system by increasing the release of VIP (122). Thus, 40 Hz sensory stimulation may influence glymphatic function through similar neurovascular coupling mechanisms. This influence has the potential to reduce α-synuclein aggregation and thereby treat PD, a possibility that was confirmed in animal experiments by Liu et al. Using a PD mouse model, they reported that 40 Hz audiovisual combined stimulation activated multiple cortical areas and induced gamma oscillations in mice. Moreover, it significantly reduced the deposition of phosphorylated α-synuclein (p-α-Syn) within neurons and effectively improved both motor and non-motor symptoms in mice (123), supporting the significant clinical application potential of 40 Hz audiovisual combined stimulation in the treatment of PD.

### 4.2 Therapeutic potential of 40 Hz VS/VAS for psychiatric and neurological disorders

In animal studies, Huang et al. identified a neural pathway—the retina-ventral lateral geniculate nucleus/intergeniculate leaflet-lateral habenula (retina-vLGN/IGL-LHb) pathway—underlying the antidepressant effects of sustained bright light therapy, which suggests that flickering light holds promise for treating depression and has an anatomical basis (124). Existing research supports the therapeutic potential of 40 Hz VS for depression. In animal studies, Zhu et al. demonstrated that in mice, 40 Hz VS can specifically modulate the HDAC3/Cox1/EP2 pathway, reducing the expression of HDAC3 and Cox1 in the injured cortex and slightly downregulating EP2 expression in the amygdala. This phenomenon suppressed microglial overactivation in the injured cortex and improved synaptic plasticity in the hippocampus, enhancing long-term potentiation (LTP) and thereby reducing susceptibility to anxiety and depression in mice exposed to poststroke stress (125). Another study revealed that 40 Hz VS alleviates neuroinflammation induced by chronic stress via the IL-12-mediated cytokine production pathway, ameliorating anxiety-like and depression-like behaviors in chronically stressed mice (126). In human clinical trials, 40 Hz VS has shown some efficacy against depressive symptoms secondary to AD and mild major depressive disorder (MDD). Patients with AD who received 40 Hz VS for 4 h per day, 5 days per week, for more than 12 weeks experienced significant improvement in depressive and euphoric symptoms (127). In patients with mild MDD, treatment with 40 Hz flickering light delivered via specialized glasses for 4 weeks led to a significant improvement in depressive symptoms without adverse events, suggesting that 40 Hz light therapy could be a non-invasive and safe intervention for alleviating depressive symptoms in mild MDD (128). Sakalauskaitė et al. plan to administer daily 1-h, 6-week courses of imperceptible 40 Hz flickering light stimulation to patients with unipolar non-psychotic MDD. They aim to assess antidepressant effects, cognitive function, sleep status, and quality of life using scales such as the major depression inventory and functional recovery tools, and the results are expected to clarify the potential therapeutic value of 40 Hz VS for MDD (129).

Schizophrenia is a syndrome characterized by fundamental and distinctive distortions in thinking, perception, emotion, and self-awareness. Patients exhibit abnormal neural oscillations, which are directly linked to the core symptoms of the disease and are therefore considered a significant pathological feature (49, 130, 131). Abnormal gamma oscillations, which serve as indicators of excitation/inhibition (E/I) imbalance in patients with schizophrenia, have been applied in its diagnosis (132). Concurrently, 40 Hz tACS has been demonstrated to alleviate negative symptoms, hallucinations, and delusions and improve cognitive function in schizophrenia patients and is now widely used in clinical practice (133). Given that both flickering light stimulation and tACS can modulate brain function by inducing synchronized neural oscillations, 40 Hz audiovisual combined stimulation is suggested to hold significant application prospects for the treatment of schizophrenia (134).

Autism spectrum disorder is a neurodevelopmental disorder that is characterized by impairments in social interaction and communication, along with restricted, repetitive patterns of behavior, interests, or activities, and language deficits. Individuals with ASD exhibit disruptions in neuronal interactions within local neural networks and demonstrate abnormal patterns of gamma-band brain wave activity (135). Concurrently, patients with ASD show a reduced 40 Hz auditory steady-state response (136) and abnormal visual perceptual responses in the gamma frequency band (137). Consequently, aberrant gamma-frequency activity is considered a significant pathological feature of ASD, and modulating these abnormal gamma oscillations has emerged as a potential therapeutic strategy. Currently, 40 Hz transcranial photobiomodulation has been effectively applied in the treatment of both adults and children with ASD (138, 139). Additionally, in an animal study, Ju et al. investigated the effects of 40 Hz VS on ASD. They administered 40 Hz VS for 14 days to female mice with a 16p11.2 deletion (a genetic model of ASD) and reported that 40 Hz VS induced adenosine release in the PFC. This adenosine subsequently activated A1 receptors, leading to a reduction in hyperexcitable neuronal transmission and the number of excitatory synapses. Ultimately, this intervention improved deficits in social novelty recognition in 16p11.2 deletion female mice (140). These findings suggest that 40 Hz flickering light, as a non-invasive intervention, offers a novel direction for ASD treatment.

### 4.3 Improvement of sleep disorders with 40 Hz stimulation

Adenosine has hypnotic effects on primary insomnia, and the blockade of adenosine receptors may be a fundamental cause of sleep disorders (141–143). Moreover, 40 Hz flickering light stimulation can trigger gamma-band LFP in V1. Gamma oscillation activity in the brain is an energy-intensive process in which the energy supply depends on ATP metabolism, leading to an increase in extracellular adenosine levels (144). On the basis of this principle, Zhou et al. performed animal studies to demonstrate that, compared with other frequencies, 40 Hz VS induced the highest concentration of extracellular adenosine in V1. This elevated concentration persisted for several hours after light stimulation ceased, suggesting that the therapeutic effect of flickering light stimulation on insomnia is sustainable. The mechanism may involve 40 Hz activation of neurons in the V1 region, inducing intracellular accumulation of adenosine, which is then transported extracellularly via equilibrative nucleoside transporter 2 (ENT2), thereby inhibiting neuronal activity and promoting sleep. Notably, the efficacy of this treatment for insomnia was also confirmed in a study involving 49 children with insomnia (143, 145, 146).

Furthermore, sleep disturbances are highly prevalent in various neurodegenerative diseases and may even contribute to driving their pathological processes, particularly in the early stages (76). Patients with AD often experience circadian rhythm disruptions, and a bidirectional relationship exists between circadian rhythm disruption and AD. On one hand, circadian abnormalities are risk factors for the onset and progression of AD; on the other hand, AD pathology can further exacerbate the deterioration of circadian rhythms (147). In animal studies, Yao et al. administered 40 Hz VS to APP/PS1 transgenic mice for 1 h per day for 30 days. Following the intervention, the abnormal activity patterns of the AD mice were restored to a normal circadian rhythm. At the molecular level, light therapy increased the protein expression levels of core clock genes, including brain and muscle ARNT-like 1, circadian locomotor output cycles kaput, and period circadian regulator 2, suggesting the restoration of clock function in the suprachiasmatic nucleus (SCN) (148). With respect to circadian rhythm phase shifts and cognitive impairment induced by sleep–wake rhythm disruptions in mice, 40 Hz blue light also significantly ameliorated these effects, but only when light stimulation was administered during the subjective daytime of mice (149). The underlying mechanism may involve the activation of neurons in alertness-related brain regions, such as the thalamus–hippocampus–visual cortex pathway; the modulation of serotonin and cortisol levels; and enhanced brain excitability, thereby alleviating the central fatigue caused by sleep deprivation (150). In a human clinical trial, patients with AD received 40 Hz audiovisual stimulation for 3 months. Following the intervention, the treatment group showed a significant increase in interdaily stability, indicating more regular daily activity patterns. This improvement was associated with the alleviation of circadian rhythm disruptions in patients with AD (12). Another clinical trial demonstrated that patients with moderate AD who received a combined 40 Hz VAS for 1 h daily for more than 6 months exhibited a significant reduction in nocturnal activity duration (*p* < 0.03) and more stable rest–activity cycles, which helped them maintain their abilities in daily living (107).

### 4.4 Therapeutic potential 40 Hz VS/VAS for epilepsy

Patients with epilepsy exhibit abnormalities in neural synchronization (88), suggesting the potential for targeting neural synchrony as a treatment approach. Currently, transcranial direct current stimulation and transcranial magnetic stimulation (TMS) are applied in clinical treatments (151). In animal studies, to investigate whether 40 Hz VS is similarly effective against epilepsy, Tinston et al. induced seizures in 5xFAD mice and their WT littermates using an amygdala kindling model and administered combined 40 Hz audiovisual stimulation. They reported that 40 Hz audiovisual stimulation delayed the onset of kindling-induced seizures and reduced the severity of the first seizure. This effect was consistent in both WT and 5xFAD mice, indicating that its efficacy is not dependent on the amelioration of Aβ pathology (152). In a human clinical trial involving 19 patients with drug-resistant focal epilepsy, Blanpain et al. demonstrated that non-invasive 40 Hz VAS effectively reduced interictal epileptiform discharges. This approach has shown particular potential for the treatment of focal epilepsy associated with the visual cortex and medial temporal lobe, providing a basis for the development of “at-home adjunctive therapy devices for epilepsy” (153).

### 4.5 Ameliorative effects of 40 Hz VS/VAS on brain injury

Moreover, 40 Hz VS/VAS demonstrates therapeutic potential for treating brain injury conditions such as stroke, traumatic brain injury, and chemotherapy-related cognitive impairment. With respect to animal research, Zheng et al. applied flickering light stimulation at different frequencies and time points to mouse models of cerebral ischaemia. They reported that early intervention with 40 Hz VS, initiated 2 h after modeling, upregulated regulator of G protein signaling 12 (RGS12) protein and enhanced its interaction with N-type calcium channels, thereby promoting presynaptic neurotransmitter release, restoring synaptic plasticity in the CA3-CA1 pathway, rescuing hippocampal low gamma oscillations, and ultimately protecting neurons and improving cognitive function (82). In a mouse model of chronic cerebral hypoperfusion induced by unilateral common carotid artery occlusion, 40 Hz flickering light improved short-term memory and spatial working memory deficits by mitigating neuroinflammation and modulating the expression of cognition-related genes (such as Arc and Per2), protecting frontal lobe and hippocampal neurons from ischemic damage (125). Furthermore, poststroke complications such as anxiety and motor impairment severely affect patients’ quality of life. Research has shown that cortical infarction after stroke activates microglia, which, via the HDAC3/NF-κB/Cox1 pathway, lead to excessive production of prostaglandin E_2_ (PGE_2_). This process subsequently activates the E EP2 in the amygdala. This signaling cascade results in neuronal hyperexcitability, ultimately triggering fear and anxiety. The 40 Hz GVS can alleviate poststroke anxiety by downregulating the HDAC3/Cox1/EP2 pathway, inhibiting microglial overactivation, and improving synaptic plasticity (125). With respect to human studies, a clinical trial targeting poststroke limb motor impairment is in preparation. Researchers plan to treat patients with acute anterior circulation ischemic stroke using combined 40 Hz audiovisual stimulation for 25 min daily for 14 days. The therapeutic effect will be assessed using the Fugl-Meyer Assessment Scale and Brunnstrom stages of motor recovery, with the goal of improving poststroke limb motor dysfunction (154).

With respect to the treatment of traumatic brain injury, Wang et al. reported that in a mouse model of traumatic brain injury, low gamma oscillations in the hippocampal CA1 region were disrupted, accompanied by a significant increase in the expression of postsynaptic density protein-95. This phenomenon leads to postsynaptic overactivation and neuronal hyperexcitability, ultimately resulting in cognitive impairment. Intervention with 40 Hz VS restored low gamma oscillations in the hippocampal tissue of brain-injured mice, reduced PSD95 expression, suppressed neuronal hyperexcitability, and consequently improved cognitive function (155).

### 4.6 Therapeutic potential of 40 Hz VS/VAS for other diseases

Myelin is a dense, multilayered structure generated by oligodendrocytes that wrap around and insulate neurons, and plays a crucial role in the normal function of the central nervous system (156). Demyelination in the CNS is associated with various neurological disorders, such as multiple sclerosis, neuromyelitis optica, and acute disseminated encephalomyelitis (157). The protective effects of 40 Hz VS on myelin have been revealed in recent animal studies (111). The research team led by Li-Huei Tsai discovered that gamma entrainment using sensory stimuli has a protective effect against cisplatin-induced demyelination injury in mouse brain tissue. It not only significantly improves survival rates but also enhances cognitive function, potentially through mechanisms such as GENUS-mediated modulation of genes associated with cisplatin resistance and promotion of the survival of oligodendrocytes after chemotherapy. These improvements were observed to persist for up to 105 days after treatment, indicating potential long-term protective effects (158). Daniela et al., in a cuprizone-induced mouse model of demyelination, confirmed that 40 Hz GENUS could promote oligodendrocyte generation by upregulating the expression of proteins involved in synaptic plasticity, calcium homeostasis, and energy metabolism pathways while downregulating the expression of inflammatory factors. This process alleviated cuprizone-induced demyelination and restored axonal signal transmission function, suggesting a non-invasive, long-term, and safe potential therapeutic strategy for demyelinating diseases such as multiple sclerosis (159).

## 5 Mechanisms underlying 40 Hz VS/VAS treatment for brain disorders

**FIGURE 2:**
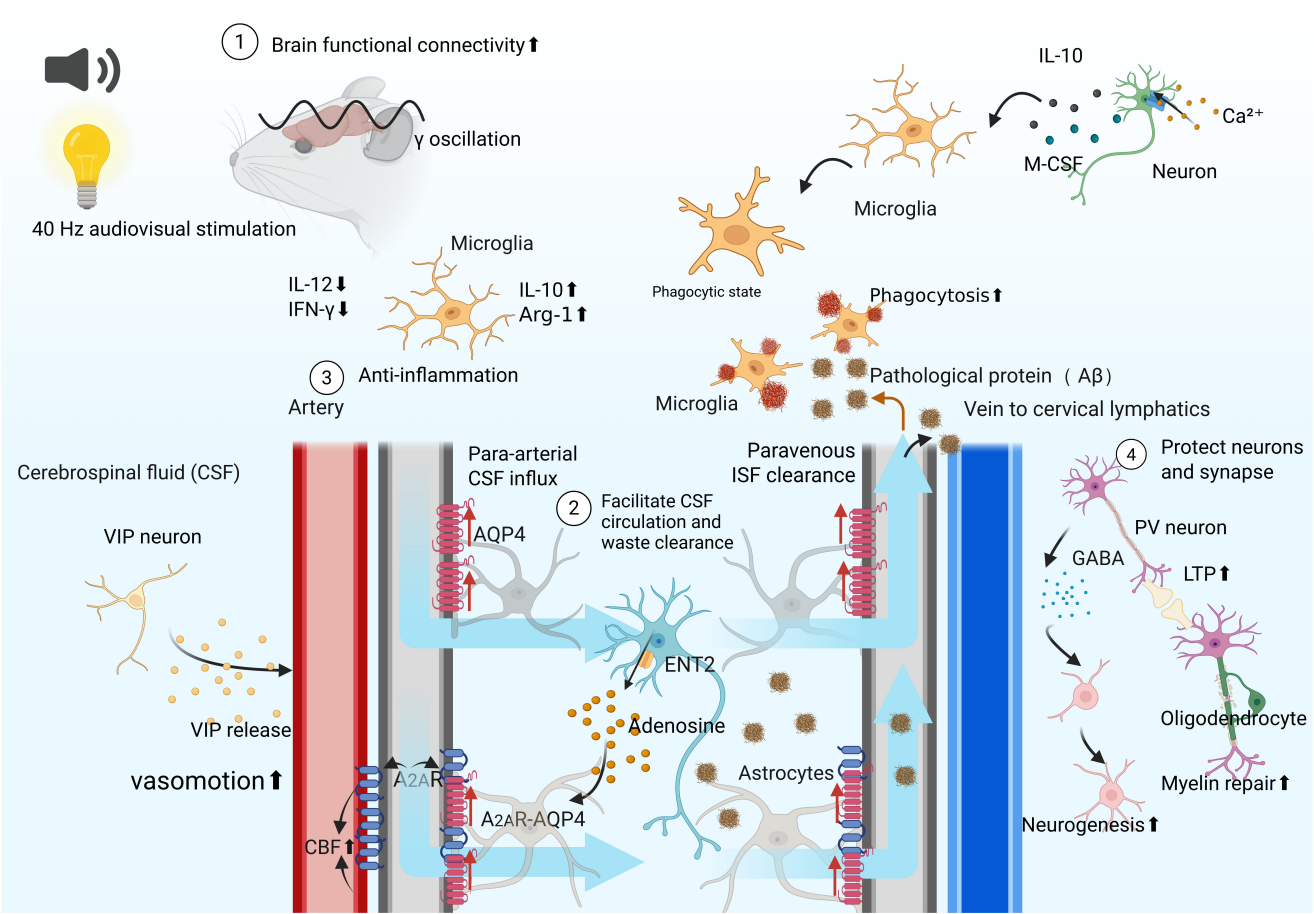
Potential Mechanisms Underlying the Therapeutic Effects of 40 Hz Stimulation in Brain Disorders. Audiovisual stimulation at 40 Hz can induce gamma oscillations in specific brain regions and enhance functional connectivity between them (12, 96, 100). It improves waste clearance capacity and reduces neuroinflammation. By modulating gene expression in various neural cells, this stimulation protects neurons and synapses (103), alters the function of glial cells (111, 112), and promotes the survival of oligodendrocytes (158). It also stimulates neurogenesis (174). Through the synergistic effects of these mechanisms, 40 Hz audiovisual stimulation may ameliorate pathological conditions and functional decline in various brain diseases. Created with BioRender.com.

As a potential non-invasive neuromodulation strategy, 40-Hz VAS exerts its effects through a complex network involving synergistic interactions at multiple levels—including neurons, glial cells, and the vascular system. Upon being perceived by sensory receptors, the stimuli are transmitted via visual and auditory pathways to various brain regions. On the one hand, they directly modulate neuronal activity; on the other hand, they indirectly influence glial cells and vascular function through bioactive molecules released by neurons. Ultimately, by impacting multiple cell types in the brain, 40-Hz stimulation exerts protective effects on neural structure and function across various dimensions, thereby offering therapeutic benefits for neurological disorders (Figure 2).

(1) Increased functional connectivity between brain regions: As the initial step in the therapeutic effect, compared with other frequencies, 40 Hz audiovisual stimulation efficiently induces synchronized neuronal firing in specific brain regions and enhances functional connectivity between regions, thereby improving information transfer efficiency. This process is likely dependent on specific neural pathways that carry visual/auditory information. Studies in AD mouse models have shown that 40-Hz stimulation enhances local oscillatory activity in the V1, CA1, SS1, and PFC regions, but also increased coordinated oscillatory activity between these regions (103). Human EEG studies have also revealed that 40-Hz stimulation improves functional connectivity in AD patients. Recent intracranial EEG (iEEG) recordings in epilepsy patients revealed that 40-Hz visual stimulation successfully entrains neural activity in advanced cognitive regions such as the hippocampus, temporal lobe, and frontal lobe, and significantly enhances synchronization between the hippocampus and the cortex (163). Research from both epilepsy and Alzheimer’s disease animal models has indicated that the efficacy of 40-Hz stimulation relies on highly specific visual pathways. Its effects are mediated by circuits involving retinal projections to the dLGN shell of the thalamus and then to PV-positive interneurons in the primary visual cortex, as well as the vLGN/IGL-Re pathway, providing a clear neural circuit basis for its therapeutic action (104, 124, 164). Enhanced interregional functional connectivity and synchronized oscillatory activity subsequently provide the necessary prerequisites of neural synchrony for subsequent broader physiological effects.

(2) Neuronal regulation and protective effects: 40 Hz VAS regulates neuronal activity and then upregulates genes involved in synaptic transmission, intracellular transport, and vesicle-mediated transport in neurons while modulating the phosphorylation levels of proteins associated with these functions (103). By regulating synaptic proteins, modulating synaptic connections and dendritic spine morphology, reducing DNA damage, and increasing the expression of neuroprotective factors, it reduces neuronal loss and mitigates degenerative pathology (103). Furthermore, 40 Hz VAS activates hippocampal PV neurons to increase GABA release (116), promoting neurogenesis. When combined with interventions such as exercise, it regulates neurogenesis through the BDNF signaling pathway (114, 115). Additionally, activated neurons release bioactive factors that modulate the function of non-neuronal cells, such as glia (113, 122, 165).

(3) Neural chemically mediated downstream systemic regulation: Following synchronized activation by 40 Hz stimulation, neurons release key neurochemical signaling molecules, which in turn drive or regulate multiple downstream physiological systems. This constitutes the core mechanism underlying its long-term therapeutic effects. Among these, adenosine and vasoactive intestinal peptide (VIP) serve as crucial messengers mediating diverse downstream outcomes. Adenosine release exerts multiple effects: in the primary visual cortex, it inhibits neuronal activity via A_1_ receptors to promote sleep (144); in the prefrontal cortex, it reduces neuronal hyperexcitability through A_1_ receptors, ameliorating social behavioral deficits in autism model mice (140); in the central amygdala, it mediates analgesia and interferes with pain memory via A2A receptors (166)and via A2A receptor signaling, it participates in driving waste clearance by the glymphatic system (113). VIP is released by activated VIPergic neurons and functions as a key signal in regulating vascular function and driving the glymphatic system. Furthermore, activated neurons can also release cytokines such as IL-10 and M-CSF, which act directly on microglia to modulate neuroimmune responses (165).

(4) Enhanced waste clearance via the glymphatic system: The enhancement of glymphatic system function is recognized as a key mechanism by which 40 Hz stimulation clears pathological proteins such as Aβ and α-synuclein, thereby exerting therapeutic effects (167, 168). This process is an important downstream effect mediated by the aforementioned neurochemical signals, VIP and adenosine. Specifically, neurons activated by 40 Hz sensory stimulation release VIP (122) while synchronized neuronal activity leads to the accumulation of adenosine, which acts via the A2A receptor signaling pathway (113). As a key component of the neurovascular unit, activated astrocytes further regulate cerebral blood flow (169). Together, they act on the vascular system to increase vascular pulsatility and cerebral blood flow, and coordinately regulate the polarization of the aquaporin-4 (AQP4) water channel protein on astrocytic endfeet. These synergistic actions collectively enhance the efficiency of cerebrospinal fluid influx into the brain parenchyma along periarterial spaces, its exchange with interstitial fluid, and the subsequent clearance of metabolic waste along perivenous pathways. This effectively promotes the removal of abnormal pathological proteins, thereby addressing the pathological roots of neurological diseases. Other frequencies of stimulation are unable to produce these effects. Furthermore, 40 Hz VAS enhances the phagocytic function of microglia, increasing their capacity to engulf pathological proteins such as Aβ (111), forming an efficient waste clearance network in concert with the glymphatic system.

(5) Alleviating neuroinflammation: Notably, the increased neuronal activity induced by 40-Hz audiovisual stimulation may further influence the structure and function of microglia. This occurs by modulating neuroimmune responses and alleviating neuroinflammation, thereby contributing to improved brain health. This modulation is partially mediated by the aforementioned neurochemical signals. Specifically, upon activation by 40-Hz visual stimulation (VS), neurons release cytokines such as IL-10 and M-CSF, which act on microglia, thus prompting morphological changes in microglia and increasing their phagocytic capacity (165). Moreover, 40 Hz VS can also trigger a series of comprehensive anti-inflammatory and immunomodulatory responses, including downregulating the expression of pro-inflammatory genes (such as interferon-response genes CD40 and C1) in microglia, suppress microglial inflammatory proliferation, and mitigate neuroinflammation mediated by these cells (103). Concurrently, 40 Hz VS exerts anti-inflammatory effects by downregulating the expression of IL-12, which consequently reduces the expression of proinflammatory cytokine IFN-γ and increases the expression of anti-inflammatory cytokine IL-10 (126), and reduces the expression of caspase-1, and inhibits the activation of the NLRP3 inflammasome (170). These outcomes work together to shape an immune microenvironment conducive to neuroprotection and repair.

(6) Effects on glial cells: In addition, owing to its direct regulatory effects on neuronal activity, 40 Hz VAS indirectly affects on glial cells, not only alters the quantity and morphology of various types of glial cells but also, more importantly, regulates the expression of specific gene sets within them. With respect to oligodendrocytes, 40 Hz flickering light treatment significantly increased this cell population and myelin sheath thickness while modulating the expression of genes related to damage repair, anti-inflammatory pathways, cell survival, and mitochondrial function. This effect contributes to the survival of oligodendrocytes and the maintenance of myelin integrity (158). The regulatory effects on microglia and astrocytes, as described previously, primarily result in downstream effects associated with enhanced waste clearance and alleviated neuroinflammation. Nevertheless, a particularly pertinent question remains: beyond their roles in waste clearance and neuroinflammation, what other functions might the changes in glial cells mediate? Astrocytes provide crucial support in the brain, supplying energy to neurons, maintaining ionic homeostasis and neurotransmitter concentrations at synapses, and directly participating in the regulation of synaptic function (122). In addition to being involved in neuroimmunity, microglia are involved in critical processes such as synaptic formation and pruning and neural repair (111, 112). Oligodendrocytes, by forming myelin sheaths around neuronal axons, facilitate the conduction of neural signals. Therefore, the effects of light therapy on glial cells may lead to extensive and profound regulatory effects on brain function, extending beyond the currently focused research areas of waste clearance and inflammation modulation. The role of glial cells in the improvement of neurological disorders through light therapy warrants further in-depth exploration.

Notably, while the current literature predominantly reports the efficacy of 40 Hz stimulation, mechanistic explanations for the inefficacy of other frequencies are lacking. A central, yet not fully resolved question is as follows: why does the 40 Hz frequency exhibit relatively unique therapeutic potential? The mechanism may lie in the fact that 40 Hz has the strongest resonance effect with endogenous gamma oscillations, thereby enabling more effective neural entrainment than other frequencies (e.g., 20 or 80 Hz). Endogenous gamma oscillations are considered the neural basis of higher cognitive functions (e.g., attention and memory), and their synchronized activity is a key mechanism for effective information exchange between different brain regions (171, 172). Consequently, exogenous 40 Hz stimulation might, via the mechanism of “neural entrainment” and in a “resonant” manner, most effectively synchronize large-scale neural networks, thereby restoring rhythmic activity impaired by disease (173). The conclusion that 40 Hz stimulation demonstrates optimal entrainment efficiency is supported by multilevel evidence. First, from a neurophysiological mechanism perspective, the generation of endogenous gamma oscillations relies on the intrinsic temporal properties of fast-spiking inhibitory circuits mediated by parvalbumin-positive interneurons. Forty hertz stimulation can efficiently activate these neurons and induce strongly synchronized gamma oscillations, thus establishing it as the optimal frequency band for synchronizing neural networks (10, 11). Second, direct frequency comparison experiments have confirmed that in Alzheimer’s disease mouse models, 40 Hz sensory stimulation is significantly superior to other frequencies (e.g., 20 and 80 Hz) in reducing Aβ pathology and improving memory (103, 122). Furthermore, neurophysiological recordings indicate that although light flicker at multiple frequencies can induce oscillatory entrainment, only 40 Hz produces the most significant enhancing effect on gamma oscillations in specific brain regions (82, 103). In summary, the unique therapeutic efficacy of 40 Hz likely stems from its strongest resonance effect with gamma oscillations. Future research needs to directly test this hypothesis through systematic comparisons across frequencies and quantitative assessments of entrainment strength. The corresponding results are visually displayed in Figure 2.

## 6 Limitations of 40 Hz VS/VAS in clinical applications and corresponding countermeasures

Currently, clinical research on 40 Hz audiovisual stimulation has focused primarily on cognitive rehabilitation and has gradually demonstrated the potential for improving mood and sleep. Notably, this stimulation can target the frontal, occipital, parietal, and temporal lobes, indicating its potential value in language and motor function rehabilitation, which warrants further exploration (13). However, the clinical efficacy of 40 Hz audiovisual stimulation still requires further clarification and optimization, its safety and compliance need enhancement, and its mechanisms remain incompletely understood. The widespread clinical application and promotion of this therapy continue to face multiple challenges.

First, although multiple studies have confirmed that 40 Hz stimulation can induce 40 Hz oscillations in the cortex (particularly in V1) (111, 122),a core controversy remains regarding whether and how such exogenously induced oscillations can “entrain” endogenous gamma oscillation networks. Evidence suggests that the intrinsic rhythm of the primary visual cortex may reside in a lower frequency band (e.g., 4–10 Hz), and that exogenous 40 Hz oscillations and endogenous oscillations may coexist rather than exhibit a strict “entrainment” relationship (175). This implies that the underlying mechanisms of 40 Hz therapeutic effects may be more complex than simple “frequency-following,” potentially involving modulation of the overall excitation/inhibition balance in neural networks or the activation of specific neural pathways (such as the vLGN/IGL-Re pathway) to produce downstream effects (104, 164).

Secondly, the efficacy of 40 Hz light therapy remains controversial, and the emergence of negative findings helps to delineate the boundaries and applicable conditions of its effects to some extent. Some studies on animals have raised questions regarding the pathological improvement and cognitive enhancement effects of 40 Hz stimulation (175, 176). The study by Soula et al. revealed that neither acute nor chronic 40 Hz flickering light can induce synchronized gamma oscillations in deep brain regions in AD mouse models, nor can it consistently reduce Aβ deposition. On the one hand, the researchers believe that the idea of “inducing endogenous gamma-band oscillations to reduce AD-related pathological damage” may not be the core protective mechanism of 40 Hz therapy, and exploring other (potential) mechanisms of action remains important. On the other hand, they pointed out that the aforementioned negative results may stem from differences in the details of certain pathological detection experimental methods, and thus should not be regarded as evidence against the notion that “long-term 40 Hz audiovisual stimulation may produce beneficial effects.” A study by Bentley et al. further reported that combined 40 Hz audiovisual stimulation increased exploratory activity only in rats without improving their memory performance (177). Similar controversies also exist in human clinical trials, Ismail et al. administered 40 Hz light stimulation to patients with mild-to-moderate AD for 10 days (2 h daily) and reported no significant changes in the amyloid-β burden across all the examined brain regions, suggesting that inducing amyloid clearance in the human brain may require longer treatment durations (178). A study by Hsiung et al. on healthy volunteers revealed that a single session of 40-Hz multimodal stimulation did not increase visual thresholds or visuospatial memory (175). Philip Tseng’s group applied a single session of 40-Hz audiovisual stimulation to healthy subjects. Real-time electroencephalography (EEG) observations revealed stable 40 Hz neural entrainment during the stimulation period. However, no improvements in cognitive behavior, including visual perception, attentional network function, working memory, or long-term memory, were detected across multiple types of tasks. Single-session gamma sensory stimulation entrains real-time EEG but does not increase perception, attention, or short-term or long-term memory, suggesting that the effects of single or short-term 40 Hz interventions on cognitive improvement are limited, and that the therapeutic benefits of these interventions may depend on long-term cumulative stimulation (179). The lack of consensus regarding the efficacy of 40 Hz light therapy is primarily attributable to the absence of standardized parameters in existing therapeutic protocols. Significant variations exist among studies regarding light frequency, intensity, duration, and intervention methods. Furthermore, individual differences among patients and distinct pathological features at different disease stages may influence therapeutic outcomes. Future efforts should focus on conducting more large-scale, high-quality clinical trials with standardized protocols, with particular attention given to the following three aspects: First, in the context of existing negative results, further clarification is needed regarding the conditions under which 40 Hz stimulation yields therapeutic effects—such as optimal treatment duration and frequency. The efficacy differences between unimodal and multimodal stimulation (e.g., 40 Hz VS vs. combined 40 Hz VAS) should be systematically examined. Second, population differences must be distinguished to reveal differential effects of 40 Hz stimulation among healthy individuals, those with mild cognitive impairment (MCI), and Alzheimer’s disease (AD) patients at various disease stages. This will help identify potential predictive biomarkers and develop personalized treatment protocols. Finally, standardizing treatment parameters is essential for enhancing effect consistency, validating clinical efficacy in brain function rehabilitation (180), and elucidating the core mechanisms of action.

Although current mainstream research and views generally consider 40 Hz sensory stimulation to be safe, feasible, and well-tolerated (12, 101), some individual studies express skepticism. The activation of “narrowband gamma oscillations” induced by specific VS is a key risk factor for triggering photosensitive epilepsy (91). Exposure to flickering visible light may increase the incidence of health-related issues such as headaches and eye strain, and in cases of abnormal light sources, it may even lead to epileptic seizures (181). A clinical study using 40 Hz auditory and light therapy in patients with mild to moderate AD reported treatment-related adverse events, including tinnitus and headaches, in some participants in the treatment group (182). In an animal study investigating the efficacy of 40 Hz flickering light, AD mice exhibited aversive behavior toward the stimulation, potentially related to increased acetylcholine levels in the hippocampus, suggesting that the therapy might induce stress responses and suboptimal tolerability, necessitating further optimization of treatment parameters (175). Furthermore, in most current studies on 40 Hz flickering light efficacy, flickering is perceptible, and control groups typically receive non-flickering light. This makes eliminating the influence of participants’ subjective factors on the outcomes difficult and prevents them from meeting the requirements of a double-blind trial design (12, 100). This methodological limitation may also be a primary factor contributing to the increased safety and tolerability concerns.

To further optimize 40 Hz audiovisual therapy, researchers have improved aspects such as light parameters, intervention timing, and delivery methods. With respect to light parameters, technological innovations aimed at enhancing comfort have been applied in the clinical translation research on 40 Hz audio-visual stimulation. For instance, techniques such as invisible spectrum flickering and multilight source combined stimulation mask the flicker perception through methods such as alternating spectra or superimposing non-flickering light. These approaches can reduce discomfort and the risk of photosensitive epilepsy seizures, holding promise for improving patients’ adherence to long-term use (127, 183). Another study revealed that reducing brightness significantly improved comfort without affecting flicker perception or the steady-state visual evoked potential (SSVEP) response and that 40 Hz SSVEPs could be elicited across all gaze angles (184). Furthermore, flickering light within the 36–44 Hz low-gamma frequency range effectively induced responses in cortical neural activity without showing a significant frequency preference (185). These findings suggest that future therapeutic protocols could select more suitable angles, brightness levels, and frequencies, enabling the development of personalized treatment options (185). However, related research on the therapeutic principle of ISF is lacking, which might operate through mechanisms distinct from those of perceptible flicker stimulation. Therefore, the underlying mechanism of action requires further clarification. Notably, recent studies have raised questions about its actual therapeutic value. Professor Tseng explicitly noted that while such comfort-oriented designs improve tolerability, they often lead to a significant attenuation of gamma rhythm entrainment effects in electroencephalography (EEG), and whether they can still maintain sufficient biological efficacy remains to be confirmed (186). Furthermore, data from Tseng’s team demonstrated that invisible 40-Hz flicker delivered through a multilight source configuration failed to effectively entrain human EEG rhythms, directly revealing the technical limitations of such comfort-oriented stimulation paradigms (187). A major challenge in current research is the absence of consistent criteria and quantitative definitions for pivotal concepts, including “invisible flicker” and “weak entrainment.” This is compounded by marked discrepancies in key technical parameters—such as the frequency modulation range, baseline light intensity, modulation depth, and light source configurations—adopted by different research groups (184, 188). This parameter heterogeneity leads to poor reproducibility of research findings and may give rise to contradictory outcomes, highlighting the urgent need for standardizing and quantitatively defining stimulation parameters. More importantly, the “threshold model hypothesis” proposed by Tseng provides key theoretical support for interpreting these contradictions: the therapeutic effect of 40 Hz stimulation may not linearly depend on entrainment intensity but rather follows a critical physiological threshold—only when gamma entrainment reaches a specific intensity can it activate downstream biological pathways. Weak entrainment may fail to yield therapeutic benefits simply because it does not surpass this threshold (186). This hypothesis aligns closely with the fundamental operational principles of the nervous system: just as the generation of action potentials follows a threshold principle, effects associated with 40 Hz stimulation—such as vasodilation and increased metabolic activity—may also require large-scale synchronous gamma oscillations as a foundation (113, 122). Therefore, in future clinical translation, while pursuing comfort, efforts should also prioritize parameter standardization, thoroughly validate the balance between comfort and efficacy, and delineate the minimum entrainment intensity necessary for therapeutic effectiveness. This will provide a more rigorous scientific basis for technological application.

Furthermore, regarding timing, interventions during sleep have been explored for patients with AD. This approach potentially improves compliance and aligns with the double-blind requirements of clinical trials. Hainke et al. reported that 40 Hz VS applied during different sleep stages could effectively induce gamma activity without disrupting sleep quality and even enhance the subjective sleep experience (189). This observation provides a new direction for 40 Hz VS intervention: delivering flickering light stimulation during sleep to increase compliance, allowing for longer stimulation durations, and potentially improving outcomes regarding brain pathology and associated symptoms in patients. Furthermore, the emergence of new technologies has facilitated further optimization of intervention methods. One study utilized virtual reality technology to deliver 40 Hz audiovisual stimulation. This method creates an immersive and interactive environment, enhancing the perceived salience of the stimuli and the cognitive engagement of the patient. It effectively induces gamma oscillations in the brain while also improving comfort and acceptance, demonstrating good safety and tolerability (190). Additionally, this therapy allows for flexible selection of different visual scenes and sounds on the basis of individual patient needs, enabling more personalized treatment plans.

To further explore the therapeutic potential of 40 Hz light therapy, researchers have expanded its application modalities. Multifactorial combined interventions, integrating 40 Hz stimulation with exercise, cognitive tasks, and pharmacological treatments, have demonstrated superior efficacy compared with any single therapy alone. For example, incorporating simple cognitive tasks during 40 Hz VS significantly enhances the strength of gamma neural entrainment, expands its cortical coverage, and even facilitates transmission to deep brain regions such as the hippocampus and insula (191). Park et al. demonstrated in 12-month-old 3xTg-AD mice that 40 Hz flickering light promoted Aβ clearance by stabilizing gamma oscillations, whereas exercise inhibited tau phosphorylation by activating the Akt pathway. The combination of both achieved simultaneous targeting of “dual pathologies” (Aβ and tau). Furthermore, these therapies act synergistically to restore mitochondrial function, inhibit apoptosis, and promote neurogenesis (192). Triple therapy combining 7,8-dihydroxyflavone administration with concurrent 40 Hz audiovisual stimulation and exercise has yielded even more significant improvements in AD (193). These findings collectively suggest that combining 40 Hz stimulation with exercise, cognitive tasks, and/or pharmacological interventions may produce optimal therapeutic outcomes.

## 7 Conclusion and future perspectives

This article summarizes the fundamental principles of 40 Hz VS/VAS-induced gamma oscillations, the neurological disorders associated with abnormal gamma oscillations and their core pathological mechanisms. Furthermore, the therapeutic effects of 40 Hz flickering light/auditory stimulation on various neurological disorders, the limitations of this strategy in clinical applications, and existing improvement strategies are discussed. Next, potential future research directions in this field are outlined.

(1) Exploring integration mechanisms of multisensory stimulation

Multisensory stimulation is a pivotal future direction in this field. Current research has expanded from single-modality 40 Hz visual stimulation to combined visual and auditory modalities (e.g., 40 Hz audiovisual stimulation), and begun to explore the potential of somatosensory (e.g., vibrotactile) stimulation. Compared to unimodal stimulation, multimodal stimulation holds promise for converging through distinct sensory pathways onto higher-order associative cortices, potentially eliciting stronger neural ensemble synchronization or broader network modulation. Therefore, elucidating the neural circuit integration mechanisms underlying multisensory stimulation, the temporal coupling relationships between different modalities, and the specific molecular and synaptic plasticity changes thereby induced, is crucial for maximizing synergistic therapeutic effects and achieving personalized neuromodulation.

(2) Long-term efficacy and optimal treatment duration of 40 Hz sensory stimulation

Although 40 Hz flickering light and/or auditory stimulation has been shown to ameliorate symptoms of neurological disorders, the limited treatment durations and short follow-up observation periods in existing studies make it difficult to determine whether its neuroprotective and cognitive-enhancing effects are sustainable. Therefore, future studies should systematically evaluate the efficacy of different intervention durations of 40 Hz sensory stimulation and prolong follow-up periods to identify the optimal treatment duration capable of inducing lasting benefits.

(3) Personalized frequency stimulation based on individual differences

Current research focuses primarily on 40 Hz light therapy because this frequency matches the intrinsic oscillation frequency of PV interneurons (fast-spiking inhibitory neurons) in the normal brains of some individuals. In reality, the resonant frequency of the brain may vary across species, ages, and disease states. Therefore, future clinical treatments could adopt a “measure-before-treat” personalized model. This approach involves first using EEG/MEG to measure the patient’s peak gamma oscillation frequency at rest and then applying flickering light stimulation at this identified frequency or at a frequency slightly below it. In essence, the core therapeutic objective is to guide the neural oscillations of the brain back to their original, individual normal frequency rather than rigidly adhering to the so-called “universal frequency” of 40 Hz.

(4) Elucidating the cellular and molecular mechanisms of abnormal gamma oscillations to identify novel therapeutic targets

Gamma oscillations are disrupted in various neurological disorders, with interneuron dysfunction and impaired synaptic plasticity considered the primary mechanisms underlying these abnormalities. Given that PYRs and diverse glial cell types also contribute to the generation of normal gamma oscillations, these cells likely play roles in the pathogenesis of aberrant gamma activity. However, a comprehensive and systematic understanding of the cell-specific molecular mechanisms driving abnormal gamma oscillations is still lacking. Therefore, future research should focus on elucidating the contributions of multiple cell types to gamma oscillopathies and the associated molecular pathways. A deeper exploration into the mechanisms governing the generation and dynamic regulation of gamma oscillations will not only advance fundamental knowledge but also facilitate the identification of novel disease-relevant biomarkers and cellular/molecular targets. This process, in turn, will promote earlier diagnosis and pave the way for precise targeted therapies.

(5) Utilizing advanced algorithmic modeling to predict abnormal gamma oscillations

Currently, the occurrence and dynamic evolution of abnormal gamma oscillations, which are significant for the early warning and timely intervention of diseases, remain difficult to predict, with research largely confined to identifying the superficial phenomenon of these abnormalities. In the era of artificial intelligence, advancements in brain science are closely linked to the development of algorithms. Fang et al. (194) successfully replicated frequency-varying oscillation phenomena in the brain using parallel simulation algorithms for large-scale functional column-structured networks, strongly demonstrating the predictability of biological phenomena by algorithmic models. Therefore, future work should investigate the mechanisms underlying abnormal gamma oscillations, and corresponding algorithmic models for simulations should be constructed. This research is important not only for the precise treatment of diseases but also for advancing the development of brain-inspired artificial intelligence algorithms.
